# Clinical Features of Essential Tremor and its Impact on Quality of Life in Japan

**DOI:** 10.5334/tohm.1006

**Published:** 2025-05-07

**Authors:** Shohei Okusa, Toshiki Tezuka, Yoshihiro Nihei, Jin Nakahara, Morinobu Seki

**Affiliations:** 1 Department of Neurology, Keio University School of Medicine, Tokyo, Japan; 2 Parkinson’s Disease Center, Keio University Hospital, Tokyo, Japan

**Keywords:** essential tremor, Parkinson’s disease, non-motor symptom, motor symptom, quality of life

## Abstract

**Background:**

Essential tremor (ET) is primarily characterized by action tremor, but is also associated with various non-motor symptoms (NMS). However, the diagnostic relevance of NMS in ET remains unclear. This study aimed to compare NMS and motor symptoms of ET with those of Parkinson’s disease-tremor dominant type (PD-TDT) and healthy controls (HCs) and to identify the presence and diagnostic relevance of NMS.

**Methods:**

Twenty-three ET patients, 23 PD-TDT patients, and 22 HCs were enrolled. Diagnoses of ET and PD were confirmed using Movement Disorder Society (MDS) criteria and Dopamine transporter single-photon emission computed tomography. Motor symptoms, NMS and quality of life (QOL) were evaluated using validated scales, including the Clinical Rating Scale for Tremor (CRST), MDS-Unified Parkinson’s Disease Rating Scale (UPDRS), Non-Motor Symptoms Scale for Parkinson’s Disease (NMSS), Odor Stick Identification Test for Japanese (OSIT-J), and Quality of Life in Essential Tremor Questionnaire (QUEST).

**Results:**

ET patients had significantly higher NMSS total scores and MDS-UPDRS part IB scores than HCs, with more severe sleep disturbances, fatigue, and urinary problems. CRST scores were significantly correlated with QUEST scores. Logistic regression identified CRST Part B and OSIT-J as key factors distinguishing ET from PD-TDT, with 87% sensitivity and 90% specificity.

**Discussion:**

ET patients showed more severe NMS than HCs. Differentiating ET from PD-TDT requires motor and sensory assessments, highlighting the diagnostic relevance of NMS. Comprehensive evaluation is essential for accurate diagnosis and management of ET.

## 1. Introduction

Essential tremor (ET) is a movement disorder primarily characterized by an 8–12 Hz bilateral upper limb action tremor [1]. ET manifests predominantly as tremor during posture and action, although some patients also experience rest tremor, and the intensity of the tremor can range from a minor inconvenience to a major disruption in daily life. ET affects 2.5%–10% of the population, with a higher prevalence observed in males [2]. In recent years, magnetic resonance-guided focused ultrasound therapy has emerged as a novel treatment modality for ET. With the advent of new therapies, public awareness of ET has grown, and more patients are now visiting hospitals, bringing this condition back to the forefront of clinical focus.

In 2018, the International Parkinson and Movement Disorder Society (MDS) proposed the latest diagnostic criteria for ET [1]. However, accurate diagnosis remains challenging due to the lack of reliable biomarkers for ET, which can result in occasional misdiagnosis. Patients presenting with tremor may be inaccurately diagnosed with ET if other tremor-related conditions have not been sufficiently ruled out [3,4]. The latest diagnostic criteria also introduced the concept of “ET plus”, which includes patients with typical ET alongside additional features such as impaired tandem gait, questionable dystonic posturing, cognitive decline, or other mild neurological signs. However, this concept has led to some confusion, as it increases the risk that other neurodegenerative diseases with tremor, such as Parkinson’s disease (PD), might be misdiagnosed as ET-plus [1]. Additionally, the classification of ET-plus with rest tremor remains a topic of ongoing debate. While some studies suggest that rest tremor may represent a late-stage manifestation of ET, other evidence indicates that it may reflect a distinct clinical entity with lower dependence on genetic susceptibility than ET, or even a prodromal state leading to PD [5].

Dopamine transporter single-photon emission computed tomography (DAT-SPECT), which is used to visualize the loss of dopaminergic neurons in the striatum, shows normal uptake in ET, whereas a normal DAT-SPECT finding is an absolute exclusion criterion in PD [6]. Therefore, DAT-SPECT is highly useful for differentiating ET from PD. However, many previous studies have diagnosed ET based solely on clinical symptoms without performing DAT-SPECT. This is likely because many of these studies were conducted before DAT-SPECT became widely used in clinical practice, and due to the high cost of the test.

PD is a progressive neurodegenerative disorder characterized by motor symptoms such as bradykinesia, tremor, and rigidity. PD can be classified into tremor-dominant type (TDT), postural instability/gait disturbance type, and mixed type depending on the predominant symptoms [7]. In the early stages, it is sometimes difficult to clinically distinguish PD-TDT from ET based solely on symptoms [3]. Furthermore, the relationship between ET and PD remains a topic of debate, with conflicting views on whether ET can convert to PD during the clinical course [8,9,10].

Recently, it has been well recognized that PD patients exhibit a variety of non-motor symptoms (NMS), such as psychiatric symptoms, autonomic dysfunction, sensory disturbances, and sleep disorders, even in the early stages of the disease [11]. It has also been reported that NMS are more closely related to the quality of life (QOL) in PD patients than motor symptoms [12]. In addition, neurologists specializing in movement disorders often obtain diagnostic clues from prodromal NMS. Similarly, various NMS, such as cardiovascular dysfunction, sleep disturbances, mood changes, attention deficits, and hearing loss, have been reported in ET [13,14,15,16,17,18,19,20,21,22,23]. Although these NMS may provide important diagnostic clues and affect QOL in ET patients [24,25,26], their clinical significance remains unclear. In particular, it is uncertain whether NMS have distinct diagnostic value for differentiating ET from PD or impact QOL in a prognostically meaningful way. Additionally, large-scale studies are lacking, and these findings are not yet well-established.

This study aimed to evaluate the motor and non-motor features of ET patients accurately diagnosed by DAT-SPECT, compared to PD-TDT and healthy controls (HCs). We also assessed their potential utility as diagnostic markers.

## 2. Methods

### 2.1 Subjects

Twenty-three patients with ET and 23 patients with PD-TDT attending an outpatient clinic at Keio University Hospital agreed to participate in this study. The diagnosis of ET was made according to the latest MDS diagnostic criteria [1]. PD patients were diagnosed with clinically established or probable PD based on the MDS diagnostic criteria [6]. In addition, only PD patients classified as tremor-dominant type based on the predominant symptoms were recruited in this study [7]. Healthy individuals with no signs of central nervous system disorders served as an age and gender-matched control group.

### 2.2 Survey

We collected the patients’ background information, including age, gender, onset age, tremor duration, treatment duration, and the results of nuclear medicine examinations such as DAT-SPECT and123I-metaiodobenzylguanidine myocardial scintigraphy (MIBG scintigraphy), from medical records. We also administered a self-report questionnaire that we developed, in which patients were asked about any worsening of tremor while writing, eating, experiencing nervousness, or at rest; improvement with alcohol consumption; and their family history of tremor and PD. The severity of tremor was assessed using the Clinical Rating Scale for Tremor (CRST), and motor symptoms of PD were assessed with the MDS-Unified Parkinson’s Disease Rating Scale (UPDRS) Part III. Cognitive function was evaluated using the Mini Mental State Examination (MMSE), the Japanese version of the Montreal Cognitive Assessment (MoCA-J), and the clock drawing test (CDT) using the Rouleau method. NMS were assessed using the Non-Motor Symptoms Questionnaire (NMSQ), Non-Motor Symptoms Scale for Parkinson’s Disease (NMSS), and MDS-UPDRS Part IB. Olfactory function was evaluated with the Odor Stick Identification Test for Japanese (OSIT-J), hearing was assessed using the Hearing Handicap Inventory for the Elderly (HHIE), anxiety and depression were evaluated using the Generalized Anxiety Disorder-7 (GAD-7) and the Geriatric Depression Scale-15 (GDS-15), and rapid eye movement sleep behavior disorder (RBD) was assessed using the RBD Single-Question Screen (RBD1Q) [27]. Activities of daily living (ADL) were assessed using MDS-UPDRS Part II. QOL was measured with the Quality of Life in Essential Tremor Questionnaire (QUEST) for ET and the Parkinson’s Disease Questionnaire (PDQ-39) for PD.

The study was approved by the ethics committee of our institution (#20211046), and informed consent was obtained from all participants.

### 2.3 Statistical analysis

Demographic data are presented as frequencies and means (±SD). The chi-squared test was employed to analyze gender differences and the responses to the self-administered questionnaire and RBD1Q among groups. The Mann-Whitney U test was used for comparisons between two groups. The Kruskal-Wallis test with Bonferroni correction was applied for comparisons among ET, PD-TDT and HC. Effect sizes were calculated using Cohen’s d for continuous variables and odds ratios (ORs) with 95% confidence intervals (CIs) for categorical variables, to assess the magnitude of group differences in NMS.

The relationship between the QUEST score and the CRST score or MDS-UPDRS Part II was investigated using Spearman’s rank correlation coefficient. We conducted a multivariable logistic regression analysis with diagnosis (ET or PD-TDT) as the dependent variable and included factors showing significant differences between ET and PD-TDT as independent variables. A stepwise method was used for variable selection and effect sizes were reported for variables retained in the final model. Based on the obtained results, we further analyzed diagnostic performance using a receiver operating characteristic (ROC) curve and calculated sensitivity and specificity. The level of statistical significance in this study was defined as *p* < 0.05. Statistical analysis was carried out using the commercial software package SPSS 29.0. Post-hoc power analysis was performed using G*Power to assess the statistical power of our correlational analyses based on the observed effect sizes and sample size.

## 3. Results

### 3.1 Clinical variables (Table 1)

**Table 1:** Clinical characteristics of ET and PD-TDT patients and HCs.


	ET	PD-TDT	HC	*p*-VALUE

Number of subjects	23	23	22	–

Age (years old)	69.3 ± 14.7	67.6 ± 10.3	70.8 ± 4.7	0.57

Gender (male:female)	18:5	15:8	13:9	0.37

Onset age (years old)	48.2 ± 21.7	62.6 ± 10.0	NA	0.007 **

Tremor duration (years)	21.1 ± 18.9	5.1 ± 3.5	NA	<0.001 ***

Therapy duration (years)	2.8 ± 3.3	3.7 ± 2.7	NA	0.11

Nuclear medicine (n, %)	23 (100%)	23 (100%)	NA	–

DAT-SPECT (reduced/normal uptake)	0/23	22/0	NA	–

MIBG scintigraphy (reduced/normal uptake)	0/3	11/8	NA	–

QUEST	18.3 ± 13.7	NA	NA	–

PDQ-39	NA	20.4 ± 17.4	NA	–


**p < 0.01, ***p < 0.001: ET versus PD-TDT. Above values are the mean±SD, except gender (given as a ratio) and nuclear medicine (given as a number and percentage). Abbreviations: DAT-SPECT: dopamine transporter single-photon emission computed tomography; ET: essential tremor; HC: healthy control; MIBG: 123I-metaiodobenzylguanidine myocardia; NA: not available; PD-TDT: Parkinson’s disease-tremor dominant type; PDQ-39: Parkinson’s Disease Questionnaire-39; QOL: quality of life; QUEST: Quality of Life in Essential Tremor Questionnaire.

Twenty-three patients with ET, 23 patients with PD-TDT, and 22 HCs participated in the study. In the ET group, no cases exhibited dystonia, cerebellar ataxia, or parkinsonism. While no significant cognitive impairment was observed in any of the assessments, some cases presented with resting tremor. Although the presence of resting tremor qualifies these cases as ET plus, no other underlying etiology could be identified. Therefore, as the diagnosis of ET as a tremor syndrome remained valid, these cases were included in the ET group. There were no significant differences in age among the three groups, while ET patients had a significantly younger age of onset and longer tremor duration than PD-TDT patients. All 23 ET patients who underwent DAT-SPECT showed normal uptake, while all 22 PD-TDT patients who underwent DAT-SPECT had reduced uptake in the striatum. MIBG scintigraphy was performed in 19 PD-TDT patients, and 11 patients showed decreased uptake in the heart. Eight PD-TDT patients with normal uptake on MIBG scintigraphy all had a tremor duration of less than 5 years and showed reduced uptake on DAT-SPECT.

### 3.2 Non-motor symptoms (Table 2)

**Table 2:** Frequency and severity of non-motor symptoms in ET and PD-TDT patients and HCs.


	ET	PD-TDT	HC	*P*-VALUE	COHEN’S D ET VS PD	COHEN’S D ET VS HC	COHEN’S D PD VS HC

NMSQ total score	5.0 ± 4.2	6.9 ± 4.2 ^‡‡‡^	2.4 ± 3.0	<0.001	0.51	0.69	1.3

NMSS total score	19.5 ± 14.1 ^†††^	33.5 ± 23.4 ^‡‡‡^	7.6 ± 9.0	<0.001	0.72	1.0	1.5

Cardiovascular	1.0 ± 1.8	0.7 ± 1.2	0.2 ± 0.7	0.06	0.17	0.57	0.52

Sleep/fatigue	5.9 ± 4.7 ^††^	8.4 ± 5.9 ^‡‡‡^	2.0 ± 3.7	<0.001	0.43	0.94	1.3

Mood	0.6 ± 1.6	2.1 ± 4.8 ^‡‡‡^	0.05 ± 0.2	0.001	0.43	0.50	0.61

Perceptual problems	0.1 ± 0.5	0.9 ± 2.0 ^‡‡^	0.0 ± 0.0	0.02	0.51	0.37	0.61

Attention/memory	2.4 ± 3.3 ^††^	3.7 ± 4.9 ^‡‡^	1.0 ± 2.7	0.003	0.30	0.48	0.68

Gastrointestinal	1.5 ± 3.1	5.2 ± 5.7 **^, ‡‡‡^	0.7 ± 1.5	<0.001	0.78	0.34	1.1

Urinary	7.5 ± 8.9	9.4 ± 9.1	3.4 ± 3.1	0.07	0.22	0.61	0.89

Sexual function	0.1 ± 0.5	0.3 ± 0.6	0.2 ± 0.9	0.10	0.33	0.07	0.17

Miscellaneous	1.5 ± 3.5	2.9 ± 2.8 **^, ‡‡‡^	0.1 ± 0.4	<0.001	0.44	0.57	1.4

MDS-UPDRS Part IB total score	5.4 ± 4.0 ^††^	5.4 ± 3.0 ^‡‡^	2.5 ± 2.4	0.002	0,02	0.87	1.1

Sleep problems	1.2 ± 1.3	0.9 ± 1.0	0.5 ± 0.8	0.07	0.28	0.72	0.52

Daytime sleepiness	1.0 ± 0.9	1.0 ± 0.9	0.7 ± 0.8	0.31	0.0	0.50	0.50

Pain and other sensations	0.6 ± 0.9	0.5 ± 0.5	0.2 ± 0.4	0.16	0.19	0.57	0.53

Urinary problems	0.9 ± 1.1 ^†^	0.9 ± 0.9 ^‡‡^	0.2 ± 0.4	0.004	0.04	0.83	1.1

Constipation problems	0.5 ± 0.9	1.0 ± 1.0 ^‡‡^	0.4 ± 0.8	0.02	0.55	0.14	0.71

Lightheadedness on standing	0.5 ± 0.8	0.3 ± 0.5	0.1 ± 0.5	0.13	0.27	0.53	0.36

Fatigue	0.7 ± 0.7	0.8 ± 0.7	0.5 ± 0.6	0.28	0.18	0.30	0.49

MMSE	28.3 ± 1.9	29.0 ± 1.6	29.4 ± 1.0	0.09	0.35	0.70	0.34

MoCA-J	25.3 ± 3.1	26.2 ± 2.9	26.9 ± 2.5	0.17	0.27	0.54	0.26

CDT	8.9 ± 1.2^*^	9.6 ± 0.8	–	0.04	0.65	–	–

OSIT-J	8.3 ± 2.3	5.1 ± 2.8 ***^, ‡‡‡^	8.9 ± 1.9	<0.001	1.3	0.25	1.6

HHIE	4.6 ± 7.4	4.4 ± 7.1	4.2 ± 7.2	0.96	0.04	0.03	0.001

GAD-7	2.5 ± 3.3	2.7 ± 4.2	1.4 ± 3.0	0.12	0.04	0.36	0.35

GDS-15	3.8 ± 3.6	4.2 ± 4.1	2.1 ± 2.6	0.14	0.10	0.55	0.62

	ET	PD-TDT	HC	*p*-value	OR ET vs PD	OR ET vs HC	OR PD vs HC

RBD1Q (positive, n (%))	2 (8.7)	6 (26.1) ^‡^	0 (0)	0.02	3.7	–	–


*p < 0.016, **p < 0.01, ***p < 0.001: ET versus PD-TDT. ^†^*p* < 0.016, ^††^*p* < 0.01, ^†††^p < 0.001: ET versus HC. ^‡^*p* < 0.016, ^‡‡^*p* < 0.01, ^‡‡‡^*p* < 0.001: PD-TDT versus HC. Above *p*-values were adjusted using the Bonferroni method. Values represent the means (±SD). Abbreviations: CDT: clock drawing test; ET: essential tremor; GAD-7: Generalized Anxiety Disorder-7; GDS-15: Geriatric Depression Scale-15; HC: healthy control; HHIE: Hearing Handicap Inventory for the Elderly; MDS-UPDRS: Movement Disorder Society-Unified Parkinson’s Disease Rating Scale; MMSE: Mini Mental State Examination; MoCA-J: Japanese version of the Montreal Cognitive Assessment; NMSQ: Non-Motor Symptoms Questionnaire; NMSS: Non-Motor Symptoms Scale for Parkinson’s Disease; OR: Odds Ratio; OSIT-J: Odor Stick Identification Test for Japanese; PD-TDT: Parkinson’s disease-tremor dominant type; RBD1Q: Rapid Eye Movements Sleep Behavior Disorder Single-Question Screen.

In terms of overall NMS, the ET group showed a significantly higher NMSS total score and MDS-UPDRS Part IB score (Kruskal-Wallis test with Bonferroni correction, *p* < 0.001, Cohen’s d = 1.0 and *p* < 0.001, Cohen’s d = 0.87) than the HC group, indicating a large effect size. The ET group showed a significantly higher score in the sleep/fatigue subdomains of the NMSS and in the urinary problem subdomain of MDS-UPDRS part IB compared to the HC group (Kruskal-Wallis test with Bonferroni correction, *p* = 0.001, Cohen’s d = 0.94, *p* = 0.013, Cohen’s d = 0.83) indicating a large effect size. Within the sleep/fatigue subdomain of the NMSS, insomnia tended to be more severe in the ET group, although the difference was not statistically significant but indicating a large effect size (Kruskal-Wallis test with Bonferroni correction, *p* = 0.025, Cohen’s d = 2.0). Regarding cognitive function, the CDT score was significantly lower in the ET group than in the PD-TDT group (Mann-Whitney U test, *p* = 0.036, Cohen’s d = 0.65) indicating a medium to large effect size, while no significant differences were found in either the MMSE or MoCA-J scores between the two groups. The OSIT-J, HHIE, GAD-7, and GDS-15 scores did not vary significantly among the three groups.

Within the ET group, patients were further analyzed by dividing them based on the median tremor duration and age of onset. The ET patients with shorter tremor duration showed significantly more severe sleep/fatigue and mood symptoms (Supplementary Table 1). Within the sleep/fatigue subdomain, insomnia was significantly more severe in the ET patients with a shorter tremor duration. When comparing by onset age, the late-onset ET patients showed significantly higher NMSS total score (Supplementary Table 2).

The PD-TDT group had a significantly higher NMSS total score (33.5 ± 23.4) compared to the HC group (7.6 ± 9.0) (Kruskal-Wallis test with Bonferroni correction, *p* < 0.001, Cohen’s d = 1.5), indicating a large effect size. Additionally, the PD-TDT group showed a higher NMSQ total score and MDS-UPDRS part IB score and a higher frequency of RBD compared to the HC group. Olfactory impairment measured using OSIT-J was also significantly more severe in the PD-TDT group compared to both the ET and HC groups (Kruskal-Wallis test with Bonferroni correction, *p* < 0.001, Cohen’s d = 1.3 and *p* < 0.001, Cohen’s d = 1.6), indicating a large effect size.

### 3.3 Motor symptoms (Table 3)

**Table 3:** Results of motor symptom evaluation and a self-report questionnaire on tremor in patients with ET and PD-TDT.


	ET	PD-TDT	*p*-VALUE

CRST total score, mean±SD	30.9 ± 18.4	29.0 ± 12.0	0.92

CRST Part A+B, mean±SD	24.4 ± 14.8	23.7 ± 10.7	0.83

CRST Part A, mean±SD	12.8 ± 9.1	16.1 ± 7.7	0.14

CRST Part B, mean±SD	11.7 ± 6.5	7.7 ± 4.5	0.026 *

CRST Part C, mean±SD	6.4 ± 4.5	5.3 ± 2.8	0.82

MDS-UPDRS Part III, mean±SD	NA	35.8 ± 13.4	

Self-report questionnaire			

Q1. Situations with worsening of tremor			

Q1-1. Writing, n (%)	12 (52.2)	10 (43.5)	0.56

Q1–2. Eating, n (%)	11 (47.8)	5 (21.7)	0.063

Q1–3. Experiencing nervousness, n (%)	15 (65.2)	16 (69.6)	0.75

Q1–4. Resting, n (%)	2 (8.7)	9 (39.1)	0.016*

Q2-1. Drinking habits, n (%)	17 (73.9)	15 (65.2)	0.52

Q2-2. Improvement of tremor with drinking, n (%)	10 (58.8)	11 (73.3)	0.39

Q3. Family history of tremor, n (%)	9 (39.1)	3 (13.0)	0.044*

Q4. Family history of Parkinson’s disease, n (%)	4 (17.4)	3 (13.0)	0.50


*p < 0.05: ET versus PD-TDT. Abbreviations: CRST: Clinical Rating Scale of Tremor; ET: essential tremor; HC: healthy control; MDS-UPDRS: Movement Disorder Society-Unified Parkinson’s Disease Rating Scale; NA: not available; PD-TDT: Parkinson’s disease-tremor dominant type.

The PD-TDT group was more likely to be aware of resting tremor compared to the ET group (chi-squared test, *p* = 0.016). There were no significant intergroup differences in reports of tremor worsening during activities such as writing and eating, or when feeling nervous, and also no significant differences in the frequency of tremor improvement with alcohol consumption. A family history of tremor was more common in the ET group than in the PD-TDT group (chi-squared test, *p* = 0.044).

In terms of tremor severity, there was no significant difference in the total CRST scores between the ET and PD-TDT groups (Mann-Whitney U test, *p* = 0.92). The analysis of the CRST subdomain revealed no significant intergroup differences in CRST Part A+B, Part A, or Part C. However, CRST Part B showed a significant difference, with the ET group scoring higher than the PD-TDT group (Mann-Whitney U test, *p* = 0.026).

### 3.4 ADL & QOL

Comparing the ET and HC groups, the MDS-UPDRS Part II score was significantly higher in the ET group (5.7 ± 6.5) compared to the HC group (0.41 ± 0.80) (Kruskal-Wallis test with Bonferroni correction, *p* < 0.001), with specific differences in the subdomains of handwriting, hobbies and other activities, tremor, and walking and balance (Supplementary Table 3). However, there was no significant difference in the total MDS-UPDRS Part II score between the ET and PD-TDT groups.

**Figure 1:**
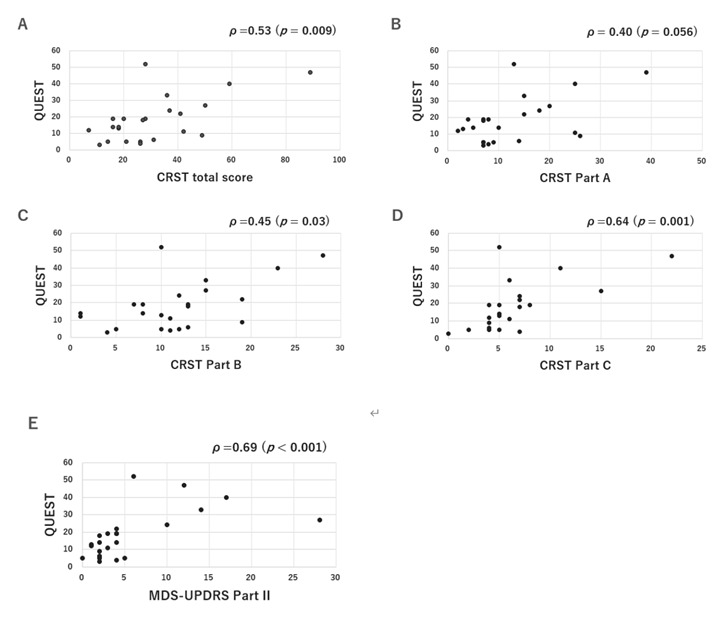
Relationship between quality of life and tremor severity **(A-D)** or activities of daily living **(E)** in ET patients.

In the ET group, Spearman’s rank correlation coefficient demonstrated a significant positive correlation between the QUEST and CRST total scores (Spearman’s rank correlation coefficient, *ρ* = 0.53, *p* = 0.009), with specific correlations noted in the CRST Part C (Spearman’s rank correlation coefficient, *ρ* = 0.64, *p* = 0.001) (Figure 1). The post hoc power for these correlations was 0.82 (CRST total) and 0.97 (Part C), respectively. Additionally, the MDS-UPDRS Part II score was strongly correlated with the QUEST score (Spearman’s rank correlation coefficient, *ρ* = 0.69, *p* < 0.001) with a post hoc power of 0.99. Conversely, in the PD-TDT group, there was no significant correlation between PDQ-39 scores and the MDS-UPDRS Part III or CRST scores.

### 3.5 Differentiation between ET patients and PT-TDT patients

**Figure 2:**
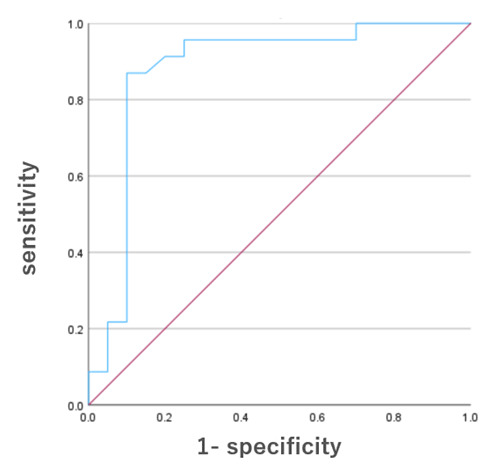
Receiver operating characteristic curves for ET and PD-TDT using CRST Part B and OSIT-J.

To differentiate between ET and PD-TDT, a multivariable logistic regression analysis was performed using the scores from CRST Part B, the gastrointestinal subdomain of NMSS, CDT, and OSIT-J as independent variables. Given the small sample size, stepwise variable selection was employed instead of the forced-entry method in order to avoid overfitting and to enhance model stability. As a result, CRST part B (OR = 0.84, 95% CI: 0.70–0.99, *p* = 0.04) and OSIT-J scores (OR = 0.55, 95% CI: 0.38–0.80, *p* = 0.002) were retained in the final model. Using these two variables, a ROC curve was constructed to evaluate the model’s discriminatory ability. The area under the curve was 0.88, with a sensitivity of 87.0% and a specificity of 90.0% (Figure 2).

## 4. Discussion

Our findings provide importantinsight into the diagnostic relevance of NMS in ET.

In this study, the ET group exhibited lower CDT scores compared to the PD group, and more severe NMS, including sleep problems, fatigue, and urinary issues, compared to the HC group. These findings confirm that NMS in ET patients are more pronounced than in healthy individuals, consistent with previous reports [16,17,18,19,20,21,22,23]. Importantly, our results also demonstrate the potential diagnostic value of certain NMS in differentiating ET from PD-TDT and HCs.

Although the precise mechanisms underlying cognitive dysfunction in ET remain poorly understood, cerebellar involvement has been increasingly emphasized, particularly through the concept of cerebellar cognitive syndrome [28,29,30]. Neuropsychological study has reported that the pattern of cognitive impairment in ET is consistent with dysfunction of the cerebello-thalamo-cortical circuitry [31]. GABA, the primary inhibitory neurotransmitter in the brain, is densely expressed in cerebellar granule cells and plays a crucial role in maintaining cerebellar circuitry. Postmortem studies have identified Purkinje cell loss and related axonal pathology in ET [32,33], which may impair GABAergic output from the cerebellum. Disruption of GABA-mediated signaling within cerebello-thalamo-cortical circuits could, in turn, contribute to the cognitive deficits observed in ET [34,35].

Although visuospatial cognitive function is known to be particularly vulnerable in PD [36], our study found that visuospatial dysfunction, as assessed by the CDT, was more severe in ET than in PD, despite similar MMSE and MoCA-J scores. This finding supports previous evidence that visuospatial assessment can help distinguish ET from PD [26]. These deficits may stem from disruptions in cerebellar afferent pathways to the posterior parietal lobes or impairments in cerebellocortical networks, leading to localized changes in the temporal and parietal cortices [37,38]. In addition, patients with ET have been reported to exhibit impairments in executive function and auditory attention/working memory [39,40], which have been attributed to the involvement of frontocerebellar circuits [41]. In this context, the pathophysiology of cognitive impairment in ET may involve not only cerebellar dysfunction due to GABAergic dysfunction, but also the involvement of the basal ganglia and widespread cortical regions.

Furthermore, cerebellar dysfunction and associated GABAergic abnormalities are thought to underline not only cognitive impairment but also sleep-related symptoms and autonomic dysfunction in ET [42,43,44]. GABA plays a key role in sleep regulation, as activation of GABAA receptors suppresses neuronal activity and promotes sleep onset [45,46]. GABA is also involved in the inhibition of bladder contraction, contributing to the regulation of urinary function [44]. A prospective, population-based study of individuals aged ≥65 years found that short sleep duration was associated with an increased risk of developing ET, suggesting that sleep disturbances may precede the onset of motor symptoms and potentially serve as an early disease marker [47]. Studies have also demonstrated that ET patients have higher Pittsburgh Sleep Quality Index scores, indicating poorer nighttime sleep quality, and that excessive daytime sleepiness (EDS) is more prevalent in ET than in HCs, particularly in patients with midline tremor [48,49,50]. Regarding urinary dysfunction, Lee et al. reported increased genitourinary symptoms in ET compared to HCs, as assessed by NMSS and the Scales for Outcomes in Parkinson’s Disease-Autonomic [22]. Consistent with these findings, our study found that insomnia, EDS and urinary dysfunction tended to be more severe in ET than HCs, with greater insomnia severity in patients with shorter tremor duration.

Additionally, olfactory dysfunction was most pronounced in the PD-TDT group, highlighting the utility of OSIT-J as a tool to differentiate PD from ET. Our multivariable logistic regression analysis demonstrated that a combination of motor (CRST Part B) and non-motor (OSIT-J) measures achieved high diagnostic accuracy in distinguishing ET from PD-TDT. While NMS in ET are complex, investigating their underlying mechanisms may enhance our understanding of the pathophysiology of ET. Moreover, the combination of motor and non-motor features, may be valuable for differentiating ET from PD-TDT.

In our study, tremor severity, as assessed by CRST, showed a strong correlation with QOL scores measured by QUEST. Although our study included a relatively small sample size, post hoc power analysis demonstrated a statistical power > 0.8 for both the CRST total score and Part C, suggesting that the observed correlations were sufficiently robust. This suggests that tremor severity remains the primary determinant of QOL impairment in ET, likely because ET tremors affect postural and kinetic tasks essential for daily functioning. On the other hand, tremor is a particularly troublesome symptom in PD, we found no correlation between tremor severity, as assessed by CRST, and QOL, as measured by PDQ-39. This is likely because PD tremors primarily occur at rest rather than during action, and other motor and NMS have a greater impact on QOL.

This study had several limitations. The sample size was small, and the study was conducted at a single center. The post-hoc power analysis revealed that the power to detect medium correlations (r = 0.30) was 0.42, suggesting a limited ability to detect small-to-medium effect sizes with the current sample size. Due to the limited sample size, we adopted a stepwise variable selection method rather than a forced-entry method in the multivariable logistic regression analysis. While this approach helps to reduce the risk of overfitting, it may also increase the risk of excluding potentially relevant variables and may affect the reproducibility of the results. Moreover, medication effects, particularly those of beta-blockers, clonazepam, and primidone, were not accounted for, and these could have influenced both motor and NMS such as blood pressure, pulse rate, and drowsiness.

To summarize, our findings provide valuable insights into the diagnostic complexity and clinical relevance of NMS in ET. Certain NMS, including cognitive impairment, sleep disturbances, and urinary dysfunction, were more pronounced in ET patients compared to HCs and may contribute to distinguishing ET from PD. Furthermore, the presence of severe NMS in some ET patients underscores the need for comprehensive and individualized assessment. Clinicians should consider NMS as potential diagnostic markers and take them into account when formulating treatment strategies.

Given the increasing recognition of NMS in ET, future studies should aim to clarify their longitudinal trajectory and clinical implications from a prognostic perspective. Longitudinal investigations are particularly needed to determine whether NMS such as cognitive impairment, sleep disturbances, urinary dysfunction progress over time in parallel with motor symptoms or follow distinct trajectories. Such studies could help elucidate the underlying pathophysiology, identify early disease markers, and inform targeted interventions. Additionally, exploring the temporal relationship between NMS and QOL deterioration may refine prognostic assessments and guide individualized patient management. Large-scale, multicenter longitudinal studies with standardized NMS assessments and neuroimaging biomarkers are warranted to advance understanding in this area.

## Additional File

The additional file for this article can be found as follows:

10.5334/tohm.1006.s1 Supplementary Tables. Tables 1–3.

## Abbreviations

ADL: activities of daily living; CDT: clock drawing test; CI: confidence interval; CRST: Clinical Rating Scale for Tremor; DAT-SPECT: dopamine transporter single-photon emission computed tomography; EDS: excessive daytime sleepiness; ET: essential tremor; GABA: gamma-aminobutyric acid; GAD-7: Generalized Anxiety Disorder-7; GDS-15: Geriatric Depression Scale-15; HC: healthy control; HHIE: Hearing Handicap Inventory for the Elderly; MDS: Movement Disorder Society; MIBG: 123I-metaiodobenzylguanidine myocardial; MMSE: Mini Mental State Examination; MoCA-J: Japanese version of the Montreal Cognitive Assessment; NMS: non-motor symptom; NMSQ: Non-Motor Symptoms Questionnaire; NMSS: Non-Motor Symptoms Scale for Parkinson’s Disease; OR: odds ratio; OSIT-J: Odor Stick Identification Test for Japanese; PD: Parkinson’s disease; PDQ-39: Parkinson’s Disease Questionnaire-39; QOL: quality of life; QUEST: Quality of Life in Essential Tremor Questionnaire; RBD: rapid eye movements sleep behavior disorder; RBD1Q: Rapid Eye Movements Sleep Behavior Disorder Single-Question Screen; ROC: receiver operating characteristic; TDT: tremor dominant type; UPDRS: Unified Parkinson’s Disease Rating Scale.
