# Supplementary material for: Clinical Features of Essential Tremor and its Impact on Quality of Life in Japan
Source: Tremor Other Hyperkinet Mov (N Y). 2025 May 7;15:21. doi: 10.5334/tohm.1006 (PMC12063571; doi:10.5334/tohm.1006)
Supplement: Supplementary Tables. — Tables 1–3. [file tohm-15-1-1006-s1.pdf]

Supplemental Table 1: Comparison of clinical features between ET with shorter tremor duration and those with longer tremor duration.

	ET with shorter tremor duration (n=12)	ET with longer tremor duration (n=11)	*p* value
Age (years old)	69.3±13.7	69.2±16.2	0.95
Gender (male:female)	9:3	9:2	0.69
Tremor duration (years)	6.8±2.3	36.7±16.3	<0.001 ***
Medicine, n (%)	β-blocker 8 (66.7)<br>primidone 0 (0.0)<br>clonazepam 3 (25.0)<br>benzodiazepine 1 (8.3)<br>zonisamide 0 (0.0)	β-blocker 9 (81.8)<br>primidone 1 (9.1)<br>clonazepam 1 (9.1)<br>benzodiazepine 1 (9.1)<br>zonisamide 1 (9.1)	0.55
CRST total score	24.1±11.2	16.5±11.1	0.09
CRST Part A	9.3±5.3	14.3±7.6	0.08
CRST Part B	9.2±4.3	7.5±5.2	0.11
CRST Part C	5.5±3.7	6.4±5.4	0.27
QUEST	14.3±8.0	8.9±1.1	0.44
MDS-UPDRS Part IB	10.0±5.0	5.7±4.5	0.71
MDS-UPDRS Part II	5.2±7.6	22.6±17.4	0.21
CDT	8.9±1.3	3.9±3.8	0.90
NMSQ	5.8±4.3	17.7±14.3	0.24
NMSS	23.5±15.8	17.7±14.3	0.21
Cardiovascular	1.4±2.4	0.45±0.52	0.93
Sleep/fatigue	8.3±4.9	3.5±2.7	0.009 **
Mood	1.2±2.0	0.0±0.0	0.02 *
Perceptual problems	0.0±0.0	0.27±0.65	0.13
Attention/memory	2.8±4.2	2.0±2.0	0.95
Gastrointestinal	1.8±3.7	1.3±2.3	0.56
Urinary	6.3±6.1	8.8±11.4	0.62
Sexual function	0.1±0.3	0.2±1.7	0.90
Miscellaneous	1.8±4.5	1.2±1.7	0.61

Values represent the mean (±SD), except for gender and medicine

*$p < 0.05$, **$p < 0.01$, ***$p < 0.001$

Abbreviations: CDT: clock drawing test; CRST: Clinical Rating Scale of Tremor; ET: essential tremor; MDS-UPDRS: Movement Disorder Society-Unified Parkinson's Disease Rating Scale; NMSQ: Non-Motor Symptoms Questionnaire; NMSS: Non-Motor Symptoms Scale for Parkinson's Disease; QUEST: Quality of Life in Essential Tremor Questionnaire.

Supplemental Table 2: Comparison of background on ET divided median onset age.

	Early-onset ET (n=13)	Late-onset ET (n=10)	*p* value
Age (years old)	64.4±16.4	75.5±9.5	0.07
Gender (male:female)	12:1	6:4	0.06
Onset age (years old)	32.8±13.8	68.2±10.2	<0.001 ***
Medicine, n (%)	β-blocker 10 (76.9)<br>primidone 0 (0.0)<br>clonazepam 2 (15.4)<br>benzodiazepine 1 (7.7)<br>zonisamide 0 (0.0)	β-blocker 7 (70.0)<br>primidone 1 (10.0)<br>clonazepam 2 (20.0)<br>benzodiazepine 1 (10.0)<br>zonisamide 1 (10.0)	0.23
CRST total score	33.8±22.2	27.1±12.1	0.66
CRST Part A	14.5±11.1	10.6±5.6	0.40
CRST Part B	12.4±7.9	10.7±4.0	0.98
CRST Part C	6.9±4.9	5.8±4.0	0.75
QUEST	20.2±15.9	15.9±10.6	0.76
MDS-UPDRS PartIB	2.8±3.4	6.2±3.7	0.38
MDS-UPDRS PartII	5.0±4.6	6.7±8.6	0.73
CDT	9.0±1.1	8.8±1.4	0.82
NMSQ	4.7±4.1	6.7±4.3	0.06
NMSS	9.1±1.9	27.4±15.2	0.02 *

Values represent the mean (±SD), except for gender and medicine

*$p < 0.016$, ***$p < 0.001$

Abbreviations: CDT: clock drawing test; CRST: Clinical Rating Scale of Tremor; ET: essential tremor; MDS-UPDRS: Movement Disorder Society-Unified Parkinson's Disease Rating Scale; NMSQ: Non-Motor Symptoms Questionnaire; NMSS: Non-Motor Symptoms Scale for Parkinson's Disease; QUEST: Quality of Life in Essential Tremor Questionnaire.

Supplemental Table 3: Activities of daily living and quality of life in patients with ET and PD-TDT and HC.

	ET (n=23)	PD-TDT (n=23)	HC (n=22)	*p* value
MDS-UPDRS PartII	5.7±6.5 [†††]	7.4±4.7 [‡‡‡]	0.4±0.8	<0.001
MDS-UPDRS PartII subdomain				
Speech	0.09±0.4	0.4±0.7 [**, ‡‡]	0.0±0.2	0.005
Saliva and drooling	0.4±1.1	0.7±1.1 [‡‡]	0.0±0.2	0.01
Chewing and swallowing	0.1±0.6	0.4±0.7	0.1±0.4	0.13
Eating tasks	0.4±0.7	0.6±0.5 [‡‡‡]	0.0±0.0	<0.001
Dressing	0.3±0.7	0.6±0.7 [‡‡‡]	0.0±0.0	0.001
Hygiene	0.2±0.5	0.4±0.5 [‡‡]	0.0±0.0	0.01
Handwriting	0.9±1.0 [†††]	0.8±0.7 [‡‡‡]	0.0±0.0	<0.001
Doing hobbies and other activities	0.7±1.0 [††]	0.7±0.9 [‡‡‡]	0.0±0.0	<0.001
Turning in bed	0.3±0.9	0.3±0.5	0.1±0.3	0.29
Tremor	1.5±0.9 [†††]	1.3±0.5 [‡‡‡]	0.0±0.0	<0.001
Getting out of bed	0.4±0.7	0.5±0.6 [‡‡]	0.0±0.2	0.005
Walking and balance	0.6±1.2	0.6±0.5 [‡‡‡]	0.0±0.2	<0.001
Freezing	0.0±0.0	0.2±0.4 [*, ‡]	0.0±0.0	0.02

*$p < 0.016$, **$p < 0.01$: ET versus PD

[†]$p < 0.016$, [††]$p < 0.001$: ET versus HC

[‡]$p < 0.016$, [‡‡]$p < 0.01$, [‡‡‡]$p < 0.001$: PD-TDT vs HC.

Above values represent the means (±SD). *p*-value was adjusted using Bonferroni method.

Abbreviations: ET: essential tremor; HC: healthy control; MDS-UPDRS: Movement Disorder Society-Unified Parkinson's Disease Rating Scale; PD-TDT: Parkinson's disease-tremor dominant type.
